# Supplementary material for: Ouabain Reverts CUS-Induced Disruption of the HPA Axis and Avoids Long-Term Spatial Memory Deficits
Source: Biomedicines. 2023 Apr 14;11(4):1177. doi: 10.3390/biomedicines11041177 (PMC10135583; doi:10.3390/biomedicines11041177)
Supplement: Supplementary file 1 [file biomedicines-11-01177-s001.zip › biomedicines-2247828-supplementary.pdf]

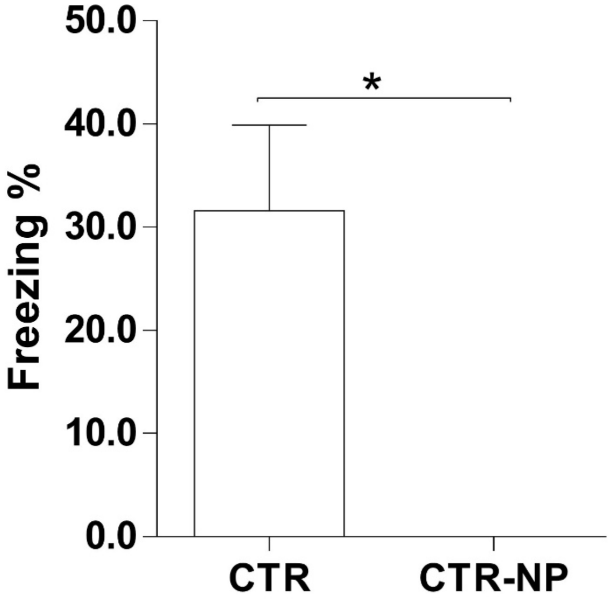

Supplementary Figure S1. Unpaired arena test. Results of analysis of behaviour in the unpaired test, where the CTR animals presented lower freezing when packed in the unpaired arena (n=4-10). Data are presented as mean ± SEM. *$p < 0.05$ when compared CTR x CTR-NP. (t-student test was performed or two-way ANOVA followed by Newman-Keuls post hoc test revealed a significant).
